# When pH comes to the rescue

**DOI:** 10.7554/eLife.62022

**Published:** 2020-09-11

**Authors:** Davi Gonçalves, Alec Santiago, Kevin A Morano

**Affiliations:** 1 Department of Microbiology and Molecular Genetics, McGovern Medical School at UTHealthHoustonUnited States; 2 MD Anderson UTHealth Graduate School of Biomedical Sciences, University of TexasHoustonUnited States

**Keywords:** heat shock, stress response, Hsf1, pH, yeast, *S. cerevisiae*

## Abstract

In starving yeast exposed to thermal stress, a transient drop in intracellular pH helps to trigger the heat shock response.

**Related research article** Triandafillou CG, Katanski CD, Dinner AR, Drummond DA. 2020. Transient intracellular acidification regulates the core transcriptional heat shock response. *eLife* **9**:e54880. doi: 10.7554/eLife.54880

Who has never had a stressful day at work? In crisis mode, we typically ensure professional survival by dropping everything and redirecting all our resources to the most important tasks, even enlisting specialized support staff to get the job done. From yeast to humans, most eukaryotic cells adopt the same strategy. When exposed to physiological stressors that may prevent their proteins from folding correctly, they call on molecular chaperones that can recognize damaged or misfolded proteins and assist in their removal or repair. In particular, events such as extreme heat, starvation or toxic substances can switch on Hsf1, the transcription factor which controls the genetic program that coordinates the creation of chaperones (Verghese et al., 2012).

This program, called the heat shock response, is shut down in the absence of stress. But how do cells then ‘know’ when to induce it? Three decades ago Betty Craig and Carol Gross of the University of Wisconsin-Madison proposed that, directly or indirectly, the trigger would involve misfolded proteins interacting with chaperones, in particular one known as Hsp70 (Craig and Gross, 1991). Recent work has largely validated this hypothesis: Hsp70 binds to Hsf1 to block its activity, until the concentration of misfolded proteins rises so much that they pull Hsp70 away from Hsf1 (Zheng et al., 2016; Krakowiak et al., 2018; Peffer et al., 2019). In this process, juvenile proteins that are just being translated act as the main Hsp70 trigger, as they are exquisitely sensitive to environmental changes and tend to misfold easily (Masser et al., 2019). Yet, starving cells – in which translation is strongly reduced – can still mount a modest heat shock response, suggesting that this neat and tidy model is actually incomplete. Now, in eLife, Allan Drummond and colleagues at the University of Chicago – including Catherine Triandafillou as first author – report a new, translation-independent pathway that triggers the heat shock response in the yeast species *Saccharomyces cerevisiae* (Triandafillou et al., 2020).

This pathway relies on the fact that elevated temperatures lead to a temporary drop in the pH of cells (Weitzel et al., 1985). To investigate how acidification is linked to the heat shock response, the team developed sophisticated and sensitive methods for monitoring ‘live’ both the pH and products of the heat shock response inside individual yeast cells. This was achieved by harnessing fluorescence readouts reported by flow cytometry (Franzmann and Alberti, 2019). In addition, the pH of the cells was ‘clamped’ at specific values.

Together, these approaches confirmed that when yeast cells are suddenly exposed to a temperature of 42 °C, their internal pH drops (from approximately 7.5 to 6.8) and their heat shock response is switched on. Under normal nutrient conditions, preventing acidification had little effect on the heat shock response. However, doing so when yeast lacked glucose all but abolished the response, and the same effect was observed in cells in which translation was blocked. Crucially, RNA sequencing experiments showed that a lack of acidification only stopped the heat shock response, in particular shutting down genes controlled by Hsf1; global transcription was not affected, and neither was a parallel stress response pathway governed by the Msn2/4 transcription factors (Verghese et al., 2012). Cellular acidification did not need to take place at the same time as the thermal stress, as a post-stress pH reduction rescued the potency of the heat shock response. In addition, pH levels needed to return to their pre-stress levels for the heat shock response to be optimal, suggesting that long-term acidification may be detrimental. Finally, the team explored whether acidification was required for cell survival. Cells that could not adjust their pH during heat shock survived, but competitive growth assays showed that they entered the cell cycle more slowly, and that they were ultimately out-competed by cells that could acidify.

The work by Triandafillou et al. uncovers a mechanism that allows cells which are not actively translating to respond to thermal stress, and to persist in a population (Figure 1). While the combination of heat shock and starvation is rare in the laboratory, it is likely common for wild yeast. These organisms lie mostly dormant and starving on the surface of fruit through the day, while they are exposed to extreme swings in ambient temperature. This ‘secondary’ way to induce the heat shock response may also help yeast face the heat and starvation they encounter when accidentally ingested by fruit-eating birds. This would enable the cells to spread to new geographic areas, providing yet another means of evolutionary competitiveness for the species.

**Figure 1.**
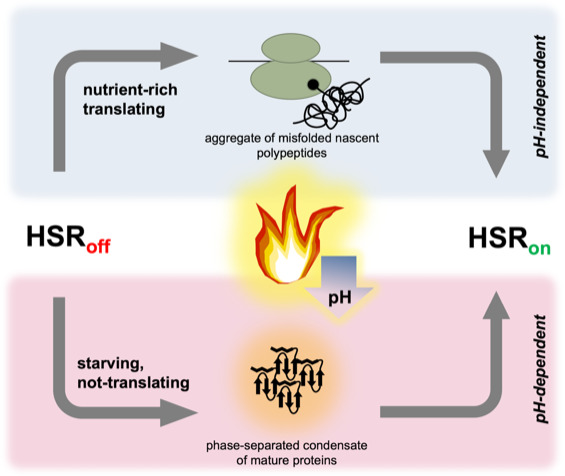
Two parallel pathways can induce the heat shock response in yeast. The heat shock response is controlled by the heat shock transcription factor Hsf1; it is repressed (HSR_off_) when conditions are stable, but rapidly induced (HSR_on_) by a high temperature. In ‘nutrient-rich’ cells (top), translation is robust and thermal stress (flame) causes a subset of nascent polypeptides to misfold and aggregate, ultimately activating Hsf1 and the heat shock response. When cells are starving (and have therefore stopped translation), the heat shock response is still induced; the trigger presumably involves mature, folded proteins assembling into phase-separated structures that can recruit chaperones. Thermal stress leads to a drop in pH (blue arrow) in all cells, but only starving cells require acidification to trigger the heat shock response.

Ironically, this discovery brings the field back full circle, to the same type of question posed over 30 years ago: what triggers this translation-independent pathway? The answer may lie in the phenomenon of phase separation, in which compartments that are not enclosed within a membrane can form inside cells to host specific biological processes. Recent work has shown that many cytoplasmic proteins can undergo phase separation to form transient assemblies that are different from the aggregates normally created by misfolding proteins (Wallace et al., 2015; Riback et al., 2017; Franzmann and Alberti, 2019). In particular, temperature and pH can control the formation of these structures. It is thus tempting to speculate that one or more such proteins, or perhaps the assemblies themselves, are recognized by Hsp70 to trigger the heat shock response. These substrates now await identification.

## References

[bib1] Craig EA, Gross CA (1991). Is hsp70 the cellular thermometer?. Trends in Biochemical Sciences.

[bib2] Franzmann TM, Alberti S (2019). Protein phase separation as a stress survival strategy. Cold Spring Harbor Perspectives in Biology.

[bib3] Krakowiak J, Zheng X, Patel N, Feder ZA, Anandhakumar J, Valerius K, Gross DS, Khalil AS, Pincus D (2018). Hsf1 and Hsp70 constitute a two-component feedback loop that regulates the yeast heat shock response. eLife.

[bib4] Masser AE, Kang W, Roy J, Mohanakrishnan Kaimal J, Quintana-Cordero J, Friedländer MR, Andréasson C (2019). Cytoplasmic protein misfolding titrates Hsp70 to activate nuclear Hsf1. eLife.

[bib5] Peffer S, Gonçalves D, Morano KA (2019). Regulation of the Hsf1-dependent transcriptome via conserved bipartite contacts with Hsp70 promotes survival in yeast. Journal of Biological Chemistry.

[bib6] Riback JA, Katanski CD, Kear-Scott JL, Pilipenko EV, Rojek AE, Sosnick TR, Drummond DA (2017). Stress-triggered phase separation is an adaptive, evolutionarily tuned response. Cell.

[bib7] Triandafillou CG, Katanski CD, Dinner AR, Drummond DA (2020). Transient intracellular acidification regulates the core transcriptional heat shock response. eLife.

[bib8] Verghese J, Abrams J, Wang Y, Morano KA (2012). Biology of the heat shock response and protein chaperones: budding yeast *Saccharomyces cerevisiae* as a model system. Microbiology and Molecular Biology Reviews.

[bib9] Wallace EW, Kear-Scott JL, Pilipenko EV, Schwartz MH, Laskowski PR, Rojek AE, Katanski CD, Riback JA, Dion MF, Franks AM, Airoldi EM, Pan T, Budnik BA, Drummond DA (2015). Reversible, specific, active aggregates of endogenous proteins assemble upon heat stress. Cell.

[bib10] Weitzel G, Pilatus U, Rensing L (1985). Similar dose response of heat shock protein synthesis and intracellular pH change in yeast. Experimental Cell Research.

[bib11] Zheng X, Krakowiak J, Patel N, Beyzavi A, Ezike J, Khalil AS, Pincus D (2016). Dynamic control of Hsf1 during heat shock by a chaperone switch and phosphorylation. eLife.

